# GlycA, a novel biomarker of systemic inflammation and cardiovascular disease risk

**DOI:** 10.1186/s12967-017-1321-6

**Published:** 2017-10-27

**Authors:** Margery A. Connelly, James D. Otvos, Irina Shalaurova, Martin P. Playford, Nehal N. Mehta

**Affiliations:** 1 Laboratory Corporation of America Holdings (LabCorp), Morrisville, NC 27560 USA; 2 National Heart, Lung, and Blood Institute, National Institutes of Health, Bethesda, MD 20892 USA

**Keywords:** Nuclear magnetic resonance spectroscopy, GlycA, Inflammation, Cardiovascular disease

## Abstract

**Background:**

GlycA is a novel spectroscopic marker of systemic inflammation with low intra-individual variability and other attributes favoring its clinical use in patients with chronic inflammatory and autoimmune diseases. GlycA is unique in its composite nature, reflecting both increased glycan complexity and circulating acute phase protein levels during local and systemic inflammation. Recent studies of GlycA from cross-sectional, observational and interventional studies have been highly informative, demonstrating that GlycA is elevated in acute and chronic inflammation, predicts death in healthy individuals and is associated with disease severity in patients with chronic inflammatory diseases such as rheumatoid arthritis, psoriasis and lupus. Moreover, following treatment with biological therapy in psoriasis, reduction in skin disease severity was accompanied by a decrease in GlycA levels and improvement in vascular inflammation.

**Conclusions:**

Collectively, these findings suggest GlycA is a marker that tracks systemic inflammation and subclinical vascular inflammation. However, larger prospective studies and randomized trials are necessary in order to assess the impact of novel therapies on GlycA in patients with chronic inflammatory conditions, which may be concomitant with cardiovascular benefits.

## GlycA is a clinical biomarker of systemic inflammation

GlycA is the name given to a particular inflammation-responsive signal in proton (^1^H) nuclear magnetic resonance (NMR) spectra of serum and plasma measured clinically [1]. The GlycA NMR signal arises largely from the *N*-acetyl methyl group protons of mobile *N*-acetyl glucosamine residues on the glycan portions of acute-phase proteins such as α1-acid glycoprotein (orosomucoid), haptoglobin, α1-antitrypsin and α1-antichymotrypsin [1–3]. GlycA levels, in units of μmol/L glycoprotein *N*-acetyl methyl groups, are associated with concentrations of IL-6, tumor necrosis factor (TNF)-α, fibrinogen, C-reactive protein (CRP; hsCRP as assessed by high-sensitivity assay), serum amyloid A (SAA), and lipoprotein-associated phospholipase A_2_ (Lp-PLA_2_) [1, 3–9]. Moreover, GlycA levels are associated with increased production of anti-microbial peptides, circulating leukocytes and neutrophil activity [3, 10]. In fact, two of the major protein contributors to the GlycA signal, α1-acid glycoprotein and haptoglobin, are synthesized in and secreted from neutrophil granules, suggesting that, besides the liver, neutrophils may be a relevant source of elevated GlycA [3, 10]. However, CRP, SAA and cytokines circulate at much lower concentrations than α1-acid glycoprotein, haptoglobin, and α1-antitrypsin, and are not highly glycosylated, therefore they contribute negligibly to the measured GlycA signal [1]. Reduced glycan mobility is another reason why some proteins with *N*-acetyl glucosamine residues, such as fibrinogen and immunoglobulin G (IgG), do not produce observable glycan NMR signals [1]. Since both positive acute phase protein levels and glycan complexity increase during inflammation, GlycA is higher in patients with acute febrile illnesses as well as chronic inflammatory diseases [3, 10, 11].

## GlycA is a marker of cardiometabolic risk

GlycA is positively correlated with body mass index (BMI), insulin resistance, markers of metabolic syndrome and the ratio of leptin to adiponectin, suggesting that in addition to being elevated in acute inflammation, levels are also raised along with adipose tissue-associated low-grade chronic inflammation [7, 12]. As such, GlycA may be a reliable biomarker of cardiometabolic risk [13]. In support of this concept, it has been reported that GlycA is associated with the presence and extent of coronary artery disease (CAD) in a secondary prevention cardiovascular (CV) cohort (CATHGEN) [14] and with incident CV events in the Women’s Health Study (WHS) [15], PREVEND [16], MESA [17] and JUPITER trial [18], independent of traditional CV disease risk factors. Notably, these associations were usually only slightly attentuated, if at all, by hsCRP implying that GlycA measures CV disease risk beyond hsCRP. GlycA is also associated with incident type 2 diabetes mellitus (T2DM) [19, 20]. Of note, the association of GlycA with incident T2DM remained statistically significant both in the WHS and PREVEND even after adjusting for traditional diabetes risk factors and hsCRP [19, 20]. Taken together, evidence suggests that GlycA may also serve as a useful biomarker for assessment of CV disease risk as well as risk of progression to T2DM.

## GlycA is associated with disease activity and CV disease in chronic inflammatory diseases

GlycA concentrations are higher in patients with autoimmune diseases such as rheumatoid arthritis (RA) [5, 21], systemic lupus erythematosus (SLE) [22, 23] and psoriasis [24]. In a cross-sectional study, GlycA was higher in RA patients compared to control subjects and was associated with increasing degree of RA disease activity [5, 21]. In this study, GlycA significantly correlated with Larsen score, a radiographic scoring of joint disease, whereas hsCRP and erythrocyte sedimentation rate (ESR) did not [5]. Moreover, GlycA was associated with coronary calcium score and known CAD in patients with RA [5]. GlycA levels were also higher in patients with SLE than matched control subjects [22]. In a cross-sectional analysis, GlycA levels were positively associated with ESR, hsCRP, E-selectin, sICAM-1 and triglycerides (TG), but not with creatinine, SLE Disease Activity Index (SLEDAI) or coronary calcium score [22]. In a separate SLE cohort, mean GlycA levels were somewhat higher in patients with high disease activity vs. patients with low or no disease activity [23]. In a longitudinal analysis, GlycA increased significantly along with increased SLEDAI [23]. Furthermore, a study in two cohorts of patients with psoriasis revealed that GlycA increased with psoriasis skin disease severity as measured by the Psoriasis Area Severity Index (PASI) [24]. GlycA was associated with non-calcified and total CAD plaque burden, as well as aortic vascular inflammation, in patients with psoriasis, independent of traditional CV risk factors including hsCRP [24]. The latter was true even after anti-TNF treatment [24]. On the other hand, after adjustment, hsCRP was not associated with total CAD or vascular inflammation in patients with psoriasis [24]. Taken together, these data support the concept that GlycA is a marker of disease activity and subclinical CV disease in patients with RA and psoriasis.

As in patients with T2DM, it is well known that patients with RA and psoriasis have higher risk of CV disease and CV events [25–31]. Traditional risk factors such as low density lipoprotein cholesterol (LDL-C) and hsCRP alone are not consistent predictors of CV disease risk in these patients. For example, LDL-C is often in the normal range, despite higher CV disease risk in RA patients [32–34]. This is often dubbed the “lipid paradox” of RA [32–34]. Moreover, addition of hsCRP to both the Framingham Risk Score (FRS) and QRISK2 equations was not associated with a significant improvement in reclassification [35]. Due to its association with CV disease in autoimmune disease patients, GlycA may represent a more effective means for assessing CV disease risk in patients with RA and psoriasis [5, 24], which remains an unmet medical need as RA and psoriasis patients continue to be at high risk of CV events and cardiovascular related mortality despite therapy [25–31].

## GlycA as a marker of mortality or prognosis

Besides being a marker of CV and T2DM risk, GlycA is also associated with mortality. Similar to IL-6 and D-dimer, GlycA was found to be related to all-cause death in MESA [17]. GlycA was also associated with colorectal cancer incidence and colorectal cancer mortality, but not breast cancer or mortality from any other cancer in the WHS [36]. GlycA was broadly predictive of hospitalization from infection and infection related mortality, even after adjusting for prevalent CV disease, diabetes, cancer, immunodeficiencies and obesity [3]. In CATHGEN, a cohort of patients undergoing cardiac catheterization, GlycA was associated with all-cause mortality, CV mortality and non-CV mortality post-catheterization [14].

## Differences between GlycA and hsCRP

As a composite biomarker, GlycA may be a better reflection of a systemic acute phase response than any single glycoprotein component [1]. For example, assays for measuring individual acute phase proteins, such as hsCRP, often exhibit high intra-individual variability [37–40]. One approach to overcome this issue is to acquire several measures of an individual marker and use the average value to assess inflammation-related risk. A second approach is to measure multiple inflammatory markers at once. GlycA, however, is already a composite biomarker that integrates the protein levels and glycosylation states of several of the most abundant acute phase proteins in serum. This allows for a more stable measure of inflammation with lower intra-individual variability for GlycA than hsCRP [1, 11]. Consequently, while guidelines recommend two serial measurements be taken at least 2 weeks apart when using hsCRP for CV risk assessment, only one measurement is necessary for evaluation of a patient’s CV risk using the GlycA test [11].

Despite similarities in disease associations, GlycA and hsCRP likely capture different aspects of the inflammatory response [10]. Epidemiological evidence for this speculation stems from the associations between GlycA and incident CV events or T2DM which were often only slightly attenuated, if at all, by the addition of hsCRP to the regression model. These results imply that GlycA measures CVD and T2DM risk beyond hsCRP [14–20]. This was also observed in the published RA study where GlycA levels, but not hsCRP, correlated with radiographic damage as measured by the Larsen score [5] as well as in the psoriasis study where GlycA, but not hsCRP, was associated with aortic vascular inflammation after adjusting for traditional CV risk factors [24]. In addition, baseline levels of both GlycA and hsCRP were found to be independent and additive markers of risk for future major adverse cardiac events (MACE), especially death and heart failure hospitalization, in patients who underwent angiography for CAD [41]. Furthermore, in most clinical studies the correlations between GlycA and hsCRP are between 0.20 and 0.50, suggesting that there is some overlap in biological processes they are detecting. This is not surprising given that CRP is an early acute phase response protein and the proteins that contribute the most to the GlycA signal (α1-acid glycoprotein, haptoglobin and α1-antitrypsin) rise later in the acute phase response [42].

## Clinical utility of the GlycA test and future perspectives

Based on the evidence gathered to date, GlycA test results may have clinical utility similar or complementary to hsCRP, fibrinogen and other biomarkers of inflammation [1, 14–20]. Therefore, GlycA may be used for the following intended uses: (1) as an aid in the identification and stratification of individuals at risk for future CV disease, (2) as an independent marker of prognosis for recurrent CV events in patients with stable coronary disease or acute coronary syndrome (ACS), (3) as an aid in the assessment of disease activity and risk of CV disease in adult RA and psoriasis patients when used in conjunction with standard clinical assessment and (4) in the evaluation of risk of progression to T2D along when considering measures of insulin resistance [43].

Of note, questions have arisen as to why “GlycA” values measured by a high-volume NMR metabolomics research platform [3, 10, 44, 45] are three- to fourfold higher than those produced by the LabCorp “clinical” GlycA assay. The origin of these differences is likely the failure of NMR metabolomics to completely separate the GlycA signal from overlapping NMR signals from proteins and very low density lipoprotein (VLDL) particles that carry the bulk of circulating triglycerides (TG). As a consequence, published correlations with TG levels are higher for the metabolomics measured GlycA (r = 0.69) than for hsCRP in the same study (r = 0.28) [45, 46]. Where TG correlations were reported for both hsCRP and “clinical GlycA”, the correlations were lower in magnitude (r ~ 0.2 to 0.4) [1, 8, 16, 22]. Adjustment for TG in multivariate regression models may alleviate some of this concern for epidemiological studies [3], but it would be difficult to use the “metabolomics GlycA” for clinical management of individual patients.

In addition to patient care applications, the potential exists for GlycA to be useful as a key biomarker in clinical studies and drug development programs. Research is underway to better characterize the relationship of inflammatory biomarkers and clinical response to various therapies. As such, GlycA measurements have been evaluated in a number of clinical trials. While GlycA levels were not significantly reduced by rosuvastatin treatment [18], they were reduced by anti-TNF therapy [24]. These studies highlight the fact that GlycA potentially provides unique information for assessing safety and monitoring efficacy of anti-inflammatory treatment. Moreover, the Phase III CANTOS study results revealed that treatment with the IL-1β antibody, canakinumab, in combination with standard of care therapy, reduced CV risk in people with a prior heart attack and inflammatory atherosclerosis [47]. This provides an additional opportunity to evaluate the relationship of various inflammatory biomarkers, including GlycA, with response to therapy. In both cases, future research may indicate the potential of GlycA to adjudicate individual response to therapy, as well as identify individuals who may benefit from further adjustment of treatment.

## Conclusions

GlycA is a composite biomarker of systemic inflammation that integrates both the protein levels and glycosylation states of several of the most abundant acute phase proteins in serum or plasma. GlycA is higher in acute and chronic inflammatory conditions and is associated with disease severity in patients with rheumatoid arthritis, systemic lupus erythematosus and psoriasis. Despite similarities in disease associations, GlycA and hsCRP likely capture different aspects of the inflammatory response. GlycA is also a marker of cardiometabolic risk that is associated with body mass index, insulin resistance, incident CV disease events and T2DM. GlycA is associated with coronary artery calcium and known CAD in rheumatoid arthritis and active vascular inflammation in psoriasis patients. Therefore, GlycA is novel a marker of CV risk that may be useful in autoimmune patients where traditional risk factors and risk scores may not accurately capture CV risk.

## Abbreviations

ACS: acute coronary syndrome
BMI: body mass index
CAD: coronary artery disease
CANTOS: Canakinumab Anti-inflammatory Thrombosis Outcomes Study
CATHGEN: CATHeterization GENetics Study
CV: cardiovascular
hsCRP: high sensitivity C-reactive protein
ESR: erythrocyte sedimentation rate
FRS: Framingham risk score
JUPITER: Justification for the Use of Statins in Prevention: an Intervention Trial Evaluating Rosuvastatin
LDL-C: low density lipoprotein cholesterol
MACE: major adverse cardiac events
MESA: Multi-Ethnic Study of Atherosclerosis
NMR: nuclear magnetic resonance
PREVEND: Prevention of Renal and Vascular End-stage Disease Study
RA: rheumatoid arthritis
SAA: serum amyloid A
SLE: systemic lupus erythematosus
SLEDAI: SLE disease activity index
T2DM: type 2 diabetes mellitus
TG: triglycerides
TNF: tumor necrosis factor
VLDL: very low density lipoprotein
WHS: Women’s Health Study
